# Radiologic maturation and tunnel changes after all-inside versus standard hamstring ACL reconstruction: a prospective comparative study

**DOI:** 10.1007/s11547-026-02177-1

**Published:** 2026-02-21

**Authors:** Alessandro Carrozzo, Edoardo Monaco, Nicola Carlo Bianco, Etienne Cavaignac, Edoardo Gaj, Riccardo Di Niccolò, Alessandro Annibaldi, Adnan Saithna, Nicola Maffulli

**Affiliations:** 1 Dipartimento di Scienze della Vita, Della Salute e delle Professioni Sanitarie, Link Campus University, Rome, Italy; 2 Orthopedic Surgery Department, Università Degli Studi “La Sapienza”, Azienda Ospedaliero-Universitaria Sant’Andrea University Hospital, Via Di Grottarossa 1035-1038, 00189 Rome, Italy; 3 Radiology Department, Ospedale San Pietro Fatebenefratelli, Rome, Italy; 4 Department of Orthopaedic Surgery, Hôpital Pierre Paul Riquet, CHU de Toulouse, Toulouse, France; 5 Orthopedic Surgery Department, Ospedale Israelitico, Rome, Italy; 6 Orthopedic Surgery Department, Ospedale San Paolo, Civitavecchia, Italy; 7 AZBSC Orthopaedics, Scottsdale, AZ USA

**Keywords:** ACL reconstruction, All-inside, MRI, Signal-to-noise quotient, Tunnel widening, Graft maturation

## Abstract

**Purpose:**

To compare MRI-based graft maturation and tibial tunnel behavior in all-inside anterior cruciate ligament (ACL) reconstruction using either a quadrupled semitendinosus (ST4) graft or a standard doubled semitendinosus–gracilis (DSTG) technique and to assess mid-term clinical outcomes.

**Materials and methods:**

This is a single-center prospective comparative cohort with sequential, non-randomized allocation. Primary imaging endpoints were graft signal-to-noise quotient (SNQ, PD-FS) and tibial tunnel cross-sectional area (CSA) change; clinical endpoints included PROMs and laxity at ≥ 24 months. Reliability was assessed with two independent evaluators and reported using ICC (two-way random, absolute agreement) with 95% CIs.

**Results:**

Forty patients (*n* = 20 per group) completed MRI at 12 months and clinical follow-up at 24 months. SNQ was similar between groups (ST4 1.94 [95% CI 1.28–2.60] vs DSTG 2.84 [1.93–3.75]; difference − 0.90 [− 1.99 to 0.19], *P* = 0.39). Tibial tunnel widening was lower with ST4 (45.5% [23.7–67.3]) versus DSTG (106.7% [78.3–135.1]); difference − 61.2% [− 95.8 to − 26.6]; *P* = .02. PROMs and laxity were comparable.

**Conclusions:**

AI-ST4 and DSTG ACL reconstruction demonstrated similar graft maturation at 12 months, with less tibial tunnel widening after AI-ST4. At 24 months, clinical outcomes were equivalent between groups; although SNQ and tibial tunnel CSA demonstrated good reproducibility, these imaging differences were not demonstrably associated with functional superiority.

## Introduction

For anterior cruciate ligament (ACL) reconstruction, the standard hamstring construct, a doubled semitendinosus–gracilis (DSTG) autograft fixed with a femoral suspensory button and a tibial interference screw, remains widely used [1]. In parallel, the all-inside (AI) technique that employs two “half tunnels” and a single quadrupled semitendinosus (ST4) graft secured by adjustable-loop cortical buttons on both sides has gained popularity because it preserves bone stock, avoids harvesting of the gracilis tendon, and provides a larger-diameter graft with comparable mechanical strength [2–4].

After ACL reconstruction (ACLR), the tendon graft initiates a progressive biological maturation process overall known as ligamentization, which affects both the intraarticular and intratunnel segments of the graft [5–8].

This complex transformation occurs over several months and progresses through overlapping histologic phases, namely an initial inflammatory stage, a proliferative stage marked by granulation tissue and fibroblast activity, and a final remodeling stage characterized by reduced vascularity and increased collagen organization [9–13].

Magnetic resonance imaging (MRI) is the most suitable noninvasive modality for assessing postoperative graft evolution [8, 14]. In addition to morphological and signal intensity evaluation, MRI provides quantitative parameters such as the signal-to-noise quotient (SNQ) and cross-sectional area (CSA). These imaging biomarkers correlate with histological and biomechanical properties of anterior cruciate ligament (ACL) graft remodeling [8, 15–17].

Current MRI data comparing the imaging evolution of all-inside ST4 and DSTG constructs remain limited and partially conflicting [18–21]. Despite theoretical concerns regarding suspensory fixation such as “bungee” and “windshield wiper,” micromotion effects that may impair tunnel healing, the AI approach offers potential advantages, including elimination of intratunnel hardware and maximized graft–bone contact area [22, 23].

The aim of this study was to compare MRI-based graft incorporation, tunnel behavior, and clinical outcomes at 12- and 24-month follow-ups between the AI with ST4 graft and traditional DSTG ACLR techniques. It was hypothesized that the AI-ST4 construct would exhibit graft signal characteristics that are comparable and result in significantly less tibial tunnel enlargement compared with the DSTG technique, while yielding similar clinical outcomes.

## Methods

This single-center, prospective comparative study was approved by our institutional review board.

Eligible participants were sequentially assigned to undergo ACLR with either a quadrupled semitendinosus graft fixed by dual adjustable-loop buttons (ST4 group) or a doubled semitendinosus–gracilis graft fixed by hybrid suspensory/interference screw technique (DSTG group). Allocation was sequential and non-randomized, determined by surgeon scheduling and implant availability.

During the study period, from January to December 2022, 123 ACL reconstructions, with different techniques and surgical timings, were performed in our setting. The inclusion criteria were skeletally mature patients, age between 15 and 50 years, chronic (> 1 month from injury) ACL tear confirmed by physical examination and preoperative MRI, healthy contralateral knee, and no prior injuries to the affected knee. Exclusion criteria were multi-ligament injuries, cartilage lesions or signs of osteoarthritis (≥ stage 2 according to the Outerbridge classification), and body mass index (BMI) greater than 30. Written consent to participate in the study was obtained from all patients.

### Endpoints

#### Postoperative MRI examination

MRI scans were performed 12 months after surgery on a 1.5-Tesla scanner (Siemens Maestro Sonata) with a dedicated eight-channel knee coil.

The knee was examined in a supine extended position.

Standard MRI knee protocol was as follows: sagittal proton-density weighted fat-suppressed (PD-FS) sequence (repetition time 1950 ms, echo time 41 ms, field of view 160 mm, slice thickness 3 mm); sagittal T1-weighted turbo-spin echo (TSE) sequence (repetition time 538 ms, echo time 11 ms, field of view 160 mm, slice thickness 3 mm); coronal PD-FS sequences (repetition time 1950 ms, echo time 41 ms, field of view 160 mm, slice thickness 3 mm); axial T2-weighted TSE sequence and axial oblique T2-weighted TSE sequence, oriented perpendicular to the long axis of the tibial tunnel using coronal and sagittal localizing scout images (repetition time 5000 ms, echo time 80 ms, field of view 160 mm, slice thickness 3 mm). Sagittal PD-FS and axial oblique TSE sequences were used to evaluate primary endpoints. Two independent authors, a fellowship-trained musculoskeletal radiologist (blinded) and a sports knee surgeon (blinded), performed all measurements; disagreements were resolved by consensus. Two imaging features were used to evaluate ligamentization and bone–tendon healing.

### 1) Signal-to-noise quotient

SNQ was calculated following the method of Weiler et al. and subsequent MRI studies on ACL graft maturation. [10, 18] Three circular regions of interest (ROIs, 0.05 cm^2^ each) were placed on sagittal PD-FS images at the proximal, mid-substance, and distal thirds of the intraarticular graft. The mean of these three values yielded signal intensity (SI) of the ACL graft.

A fourth ROI of identical size was centered in the mid-portion of the intact posterior cruciate ligament (PCL) on the same slice to obtain the reference signal intensity PCL SI.

**Fig. 1:**
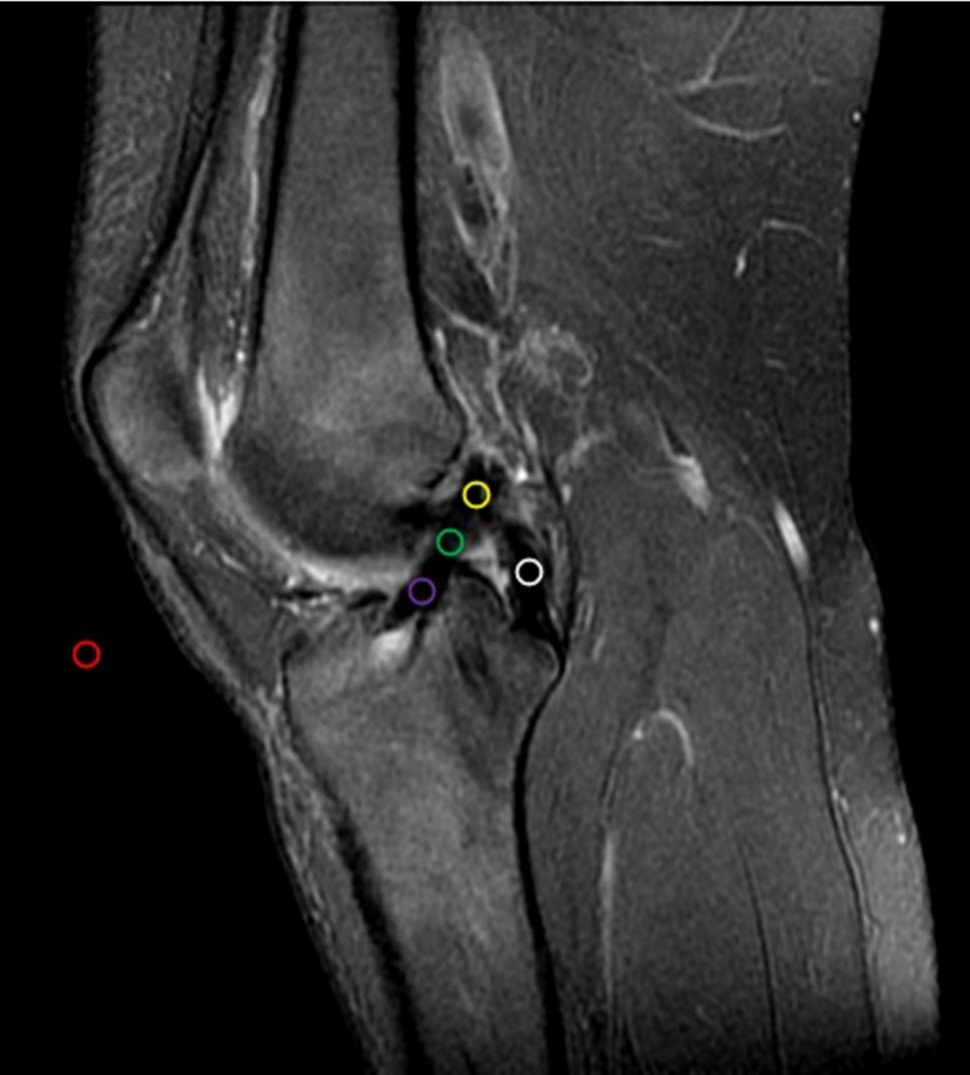
Signal-to-noise quotient (SNQ) measurement. Sagittal PDw fat-sat image showing placement of five regions of interest (ROI = 0.05 cm^2^) used to calculate SNQ: proximal (yellow), middle (green), and distal (purple) third of the ACL graft; mid-substance of the PCL (white); background ROI 2 cm anterior to the patellar tendon (red).

To characterize image noise, a background ROI was positioned 2 cm anterior to the patellar tendon, outside any anatomical structure, providing background SI. (Fig. 1)

The SNQ for each patient was then derived as:

$$SNQ = \frac{ACL\;graft\;SI - PCL\;SI}{{Background\;SI}}$$

This formulation normalizes graft signal against both physiological reference tissue (PCL) and acquisition noise, leading to a dimensionless metric that inversely correlates with graft tensile strength and integration. [15, 24, 25]

All measurements were performed twice, two weeks apart, by two independent observers using Horos® v.3.3.0 (The Horos Project, Geneva, Switzerland). Intra- and interobserver agreement was evaluated.

## 1) Cross-sectional area and tunnel widening computation

**Fig. 2:**
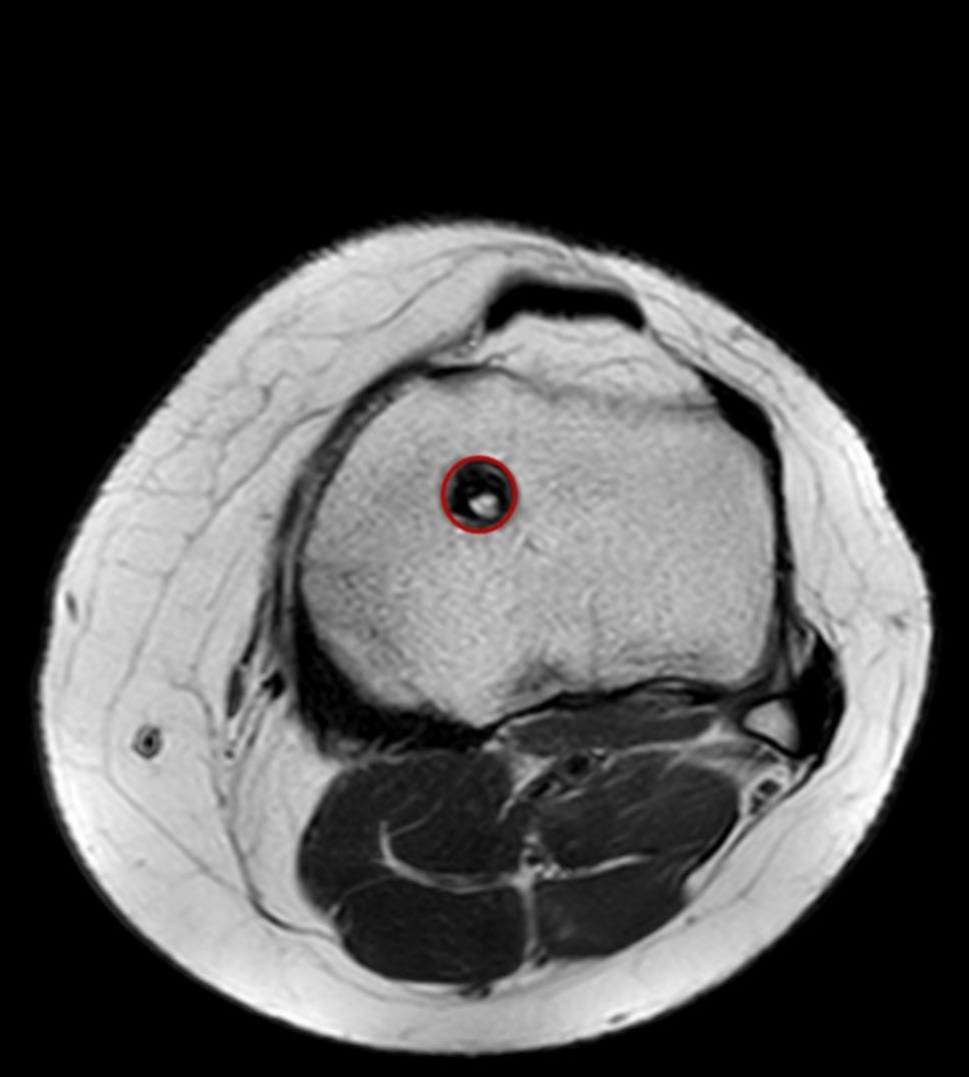
Measurement of the cross-sectional area of the tibial bone tunnel. Axial oblique T2w image showing placement of a circular region of interest with the “best-fit” method at the tibial tunnel entrance, 1 cm below the joint line.

Cross-sectional area of the tibial tunnel was measured on axial oblique T2-weighted TSE sequence, oriented perpendicular to the long axis of the tunnel. Using Horos medical image viewer for MacOS (version 3.3.0), the cross-sectional area (CSAs; in cm²) was measured on the para-axial slices nearest the entry of the tibial tunnel, which was about 1 cm under the articular surface (Fig. 2).

Cross-sectional areas change in tibial and femoral bone tunnels after ACLR were calculated with the following formula [26]:

$${\mathrm{Tunnel}}\;{\text{Widening }}\left( {{\% }} \right) = { }\frac{{{\mathrm{Measured}}\;{\mathrm{CSA}} - {\mathrm{Drilled}}\;{\mathrm{CSA}}}}{{{\mathrm{Drilled}}\;{\mathrm{CSA}}}}{ } \times 100$$

### Surgical procedures

All surgical procedures were performed by the same senior surgeon (blinded for review).

#### Doubled gracilis and semitendinosus tendons ACLR

An anatomic single-bundle technique with doubled gracilis and semitendinosus tendons ACLR technique was performed. The femoral tunnel was drilled in an outside-in fashion using a retrodrill in to obtain a 25-mm socket (Flipcutter II; Arthrex, Naples, FL, USA). The entry point of the femoral tunnel was at the center of the anatomic femoral footprint of the ACL, located midway between the resident ridge and over-the-top position, with the guide set at 100–110°. A full tibial tunnel was produced with a standard guide set at 60–65°. The graft was then shuttled from the tibial tunnel through the femoral socket and fixed on the femoral side using an adjustable-loop suspensory device (Tight-rope RT; Arthrex) and on the tibia using an absorbable interference screw (BioComposite; Arthrex), with a diameter 1 mm greater than the graft and tunnel diameter [27].

#### Quadrupled semitendinosus tendons ACLR

In the ST4 group, only the semitendinosus tendon was harvested and then quadrupled to obtain a final graft length of 70 mm. The graft was prepared in linkage with two TightRope-RT adjustable-loop cortical buttons (Arthrex) and a high strength, multi-strand, long-chain ultra-high-molecular-weight polyethylene core suture (No. 0 FiberWire; Arthrex) on each side. A 25-mm femoral socket was drilled using a retrodrill (Flipcutter II; Arthrex) in an outside-in fashion. A standard tibial guide set at 60–65° was used to produce a 25-mm tibial socket at the tibial ACL insertion using a retrodrill (Flipcutter II; Arthrex).

The graft was then introduced through the anteromedial portal using a shuttle suture on both sides and fixed first on the femoral side and then on the tibial side with the "flip-then-fill technique." [28]

### Postoperative rehabilitation

All patients followed the same nonaggressive rehabilitation protocol. A brace locked in full extension was applied for the first 2 weeks. Immediate postoperative weight-bearing as tolerated with crutches was allowed. Patients were allowed to remove the brace at 2 weeks to start exercises to recover range of motion (ROM). The brace and crutches were completely removed 4 weeks postoperatively. A muscle-strengthening program was introduced progressively and increased from the second postoperative month. Cycling and swimming were permitted at 6 weeks, jogging at 3 months, and cutting or pivoting sports at 6 months.

#### Clinical follow-up

The clinical follow-up was conducted at 2 and 6 weeks and at 3, 6, and 12 months postoperatively. In addition, all patients were recalled for outpatient evaluation between September 2024 and January 2025 for final follow-up, 24 months after surgery.

The face-to-face final evaluation was standardized and involved a physical examination conducted by a senior surgeon who had not been involved in the original procedure.

## 1) Clinical evaluation

Clinical evaluation included recording ROM and performing the Lachman and pivot shift tests. The Lachman test was graded in comparison with the contralateral side as either A (< 2 mm), B (2–4 mm), C (6–10 mm), or D (> 10 mm) [29]. The pivot shift was graded as 0 (absent), 1 (subluxation), 2 (jump), or 3 (transient lock) [29].

Also, side-to-side difference on KT-1000 arthrometer (MEDmetric; San Diego, CA, USA) maximum manual testing at 30° (mm) was evaluated. Patients were graded as A if the measurement was 0–2 mm, B if it was between 3 and 5 mm, C if it was > 5 mm, and D if it was > 10 mm.

## 1) Patient-reported outcome measures

All patients were asked to complete patient-reported outcome measures (PROMs), including the Lysholm Knee Scoring Scale and the International Knee Documentation Committee (IKDC) subjective score [29, 30]. Also, the Tegner Activity Scale at the last follow-up was recorded for each patient [31].

### Statistical analysis

All analyses were performed with the SPSS Statistics software (IBM SPSS Statistics for Macintosh, version 27.0. Armonk, NY: IBM Corp). Statistical significance was set at *P* < 0.05.

Descriptive analyses were conducted on the entire patient cohort, and descriptive statistics were calculated based on the nature of the considered criteria. To assess normality, the Kolmogorov–Smirnov test was applied. For quantitative data, this included the computation of observed and any missing values, mean, standard deviation, median, and minimum and maximum values. Qualitative data were analyzed by recording observed and missing values, in addition to the count and percentage of patients within each class. Categorical data differences between groups were explored using Chi-square or Fisher exact tests, while quantitative variables were examined through the Mann–Whitney U test or Student t test.

Cronbach alpha was employed to assess inter- and intraobserver reliability at MRI evaluations. Intra- and interobserver reliability for SNQ and tibial tunnel CSA was quantified using intraclass correlation coefficient (ICC) from two-way random-effects models, absolute-agreement definition, reported as ICC(2,1) for single-measure reliability and ICC(2,2) for the average of the two readers.

Methods and results are reported in accordance with the STROBE extension for comparative cohort studies.

## Results

### Patients

**Fig. 3:**
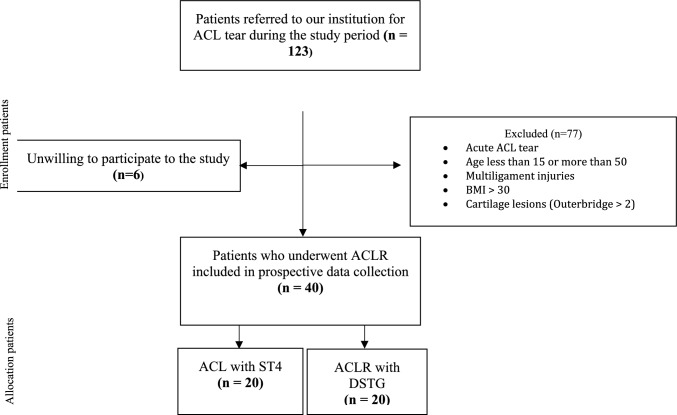
The study flowchart. ACLR, anterior cruciate ligament reconstruction; DSTG, doubled semitendinosus–gracilis tendons; ST4, quadrupled semitendinosus tendon

Of the initial 123 patients, 77 did not meet the inclusion criteria cited above. Of the remaining 46 patients, 6 did not agree to be enrolled in the study. The overall study population comprised 40 patients (20 in each group) who underwent ACLR for chronic ACL injury. No patients were lost to follow-up. The patient’s flowchart is reported in Fig. 3.

There were no significant differences between groups in terms of patient characteristics as time from injury to surgery, duration of follow-up, and preoperative Tegner activity level score, but the ACL graft diameter was statistically different (8.86 mm ± 0.41 in ST4 group; 8.2 mm ± 0.42 in DSTG group, ***P* = 0.005**).

The demographic data of two groups is shown in Table 1.

**Table 1:** Demographics of the included patients

	ST4 group (*n* = 20)	DSTG group (*n* = 20)	*p *value
Age in years, mean ± SD	31.7 ± 7.1	32.5 ± 6.7	.61
Sex (M; F), number	12; 6	10; 8	.65
BMI, mean ± SD, kg/m^2^	20.6 ± 2.1	21 ± 2.6	.37
Time between injury and surgery in days, mean ± SD	154 ± 39	121 ± 67	.18
Preoperative Tegner Activity Scale score, median ± IQR	6 ± 2	6 ± 2	n.a
Graft diameter in mm, mean ± SD	8.86 ± .0.41	8.2 ± .0.42	**.005**

IQR; interquartile range; SD, standard deviation. Boldface indicates statistical significance (***P* < .05).** n.a., not applicable (median values are equal)

### Imaging outcomes

The mean SNQ was 1.94 ± 1.41 for the ST4 group and 2.84 ± 1.95 for the DSTG group (***P* = 0.388**).

All the measured tibial tunnels underwent postoperative enlargement compared to the drilled CSA. The mean tibial tunnel widening, measured using the CSA, was 45.5% ± 46.6% for the ST4 group and 106.7% ± 60.7% for the DSTG group, resulting in a significant difference ***(P* = *0.0*2**). Imaging outcomes are presented in Table 2, and MRI sequence technical specifications in Table 3.

**Table 2:** Imaging outcomes

	ST4 (n = 20)	DSTG (n = 20)	Between-group difference (ST4 − DSTG)	*P* value
SNQ (mean ± SD)	1.94 ± 1.41 (**95% CI 1.28–2.60**)	2.84 ± 1.95 (**95% CI 1.93–3.75**)	** − 0.90 (95% CI − 1.99 to 0.19)**	.388
Tibial tunnel widening, %CSA (mean ± SD)	45.5% ± 46.6% (**95% CI 23.7%–67.3%**)	106.7% ± 60.7% (**95% CI 78.3%–135.1%**)	** − 61.2% (95% CI − 95.8% to − 26.6%)**	.020

Boldface indicates statistical significance (***P* < .005)**. CSA, cross-sectional area; SD, standard deviation; SNQ, signal-to-noise quotient

**Table 3:** MRI sequence technical specifications

Sequence (TSE unless stated)	Plane	TR (ms)	TE (ms)	FOV (mm)	Slice (mm)
PD-fat-suppressed	Sagittal	1950	41	160	3
PD-fat-suppressed	Coronal	1950	41	160	3
T1-weighted	Sagittal	538	11	160	3
T2-weighted	Axial	5000	80	160	3
T2-weighted	Para-axial	5000	80	160	3

### Intra- and interobserver reliability

The Cronbach’s alpha and the ICC were calculated to assess the intraobserver and interobserver reliability for all the MRI endpoints. Both intra- and interobserver analysis resulted in good reliability (Table 4) [32].

**Table 4:** Intra- and interobserver reliability

Measure	Alpha	Alpha 95% CI	ICC(2,1)	ICC(2,1) 95% CI	ICC(2,2)	ICC(2,2) 95% CI	p
SNQ intraobserver reliability	0.84	0.68–0.94	0.72	0.52–0.89	0.84	0.68–0.94	< 0.01
SNQ interobserver reliability	0.82	0.66–0.91	0.69	0.49–0.83	0.82	0.66–0.91	< 0.01

Intra- and interobserver reliability was calculated with Cronbach’s alpha and evaluated, according to George and Mallery [32], as unacceptable if alpha was inferior to 0.5; poor if alpha was comprised between 0.5 and 0.59; questionable, between 0.6 and 0.69; acceptable, between 0.7 and 0.79; good, between 0.8 and 0.89; excellent, greater than 0.9. Reliability was quantified with ICC, two-way random-effects, absolute agreement, with two raters. An ICC < 0.50 was considered poor, 0.50–0.75 moderate, 0.75–0.90 good, > 0.90 excellent. [32]

### Clinical outcomes

At the last follow-up, there was no significant difference between the two groups in Lysholm score, Tegner Activity Scale, and IKDC. Also, no difference was found in the grade of Lachman test, Pivot shift, and the difference in anteroposterior side-to-side translation measured at KT-1000 arthrometer (Table 5).

**Table 5:** Clinical outcomes

	ST4 group (20)	DSTG group (20)
Lysholm, mean ± SD	95 (74–100)	92 (72–100)
Postoperative Tegner Activity Scale, median ± IQR	6 (6–8)	6 (6–8)
IKDC, mean ± SD	91 (87.4–100)	94 (87.4–98.9)
Lachman test, grade		
A	13	14
B	7	6
C	0	0
D	0	0
Pivot shift test, grade		
0	16	16
1	4	4
2	0	0
3	0	0
KT-1000 in mm, mean ± SD	1.7 ± 1.2	2.1 ± 1.2

IKDC, International Knee Documentation Committee; IQR, interquartile range; SD, standard deviation. n.a., not applicable (median values are equal)

## Discussion

The main finding of the present study was that the all-inside ST4 group demonstrated comparable graft signal characteristics to the double semitendinosus and gracilis group with respect to postoperative SNQ values. In addition, no significant differences in SNQ scores between the two methods were observed (1.94 ± 1.41 for the ST4 group and 2.84 ± 1.95 for the DSTG, *P* = 0.388). The significantly lower extent of tibial tunnel widening in the AI group compared to the DSTG group (45.5% ± 46.6% for the AI group vs. 106.7% ± 60.7% for the DSTG group, *P* = 0.02) is consistent with our previous findings [33].

MRI signal intensity after ACL reconstruction can provide a noninvasive way for monitoring graft maturation and mechanical competence over time. Weiler et al. conducted a two-year animal model study demonstrating that MRI can predict both the biomechanical properties and vascularity of ACL grafts during remodeling [10]. Increased MRI signal intensity correlated negatively with load to failure, stiffness, and tensile strength. This led to the introduction of the SNQ measurements.

The literature reports SNQ values ranging from 0.078 ± 0.62 for an ST4 graft at six months to 5.49 ± 3.71 for an allograft at two years [11, 34]. In the present study, the mean SNQ values were 1.94 ± 1.41 for the AI ST4 group and 2.84 ± 1.95 for the IS DTSG group at one year. These results suggest that, given its lower SNQ values, the all-inside technique has no biomechanical disadvantage compared to DTSG fixation. Consistent with the results of the current study, Colombet et al.[40] reported better graft integration with an adjustable-loop suspensory device compared to tibial interference screw fixation in a comparative study of 109 patients.

Previous studies showed superior strength of the ST4 graft compared with the DSTG graft and that the diameter of the ST4 graft was, on average, 20% greater than that of a DSTG graft from the same individual [35, 36]. Cavaignac et al.[18] also compared SNQ between ST4 and DSTG patient groups and found no inferiority in graft integration between the techniques. This study confirms these findings but notes slightly lower SNQ values in the ST4 group. The significant difference in mean graft diameter between the current study groups (8.2 ± 0.4 mm for DSTG vs. 9 ± 0.4 mm for ST4, *P* = 0.001) may explain the observed SNQ results. Recent evidence shows that higher graft signal on magnetic resonance imaging at 12 months, indicating poorer graft healing, is associated with early anterior cruciate ligament re-tear. [37]

Historically, the majority of tunnel widening assessments have been based on plain radiographs. These methods rely on two-dimensional measurements of tunnel diameter, which are subject to notable limitations including poor tunnel visualization, magnification artifacts, and inter- and intraobserver variability [37, 38]. MRI offers the dual advantage of visualizing both tunnel configuration and graft integrity, and can also detect fluid collections or cystic changes within the tibial tunnel that may contribute to progressive enlargement. [38, 39]

The reduced degree of tibial tunnel widening seen with the all-inside technique can be attributed to several factors: first, the intentional oversizing of the screws by 1 mm more than the tunnel diameter during surgery; second, the potential benefits of retrograde drilling, which may minimize subchondral bone fragmentation and reduce synovial fluid migration into the bone tunnel [40, 41].

Conversely, concerns have been raised about the potential for tunnel widening with extracortical suspensory fixation because of graft micromotion, the "bungee cord," and "windshield wiper" effects [42]. However, recent studies have not found significant loop lengthening with adjustable-loop suspensory devices [43, 44].

The biological advantages of suspensory fixation over interference screws, such as increased tendon–bone contact area and promotion of "four-zone direct graft healing," which may explain the improved graft signal observed in the studied population [45].

Based on the present study results, ACLR using an AI-ST4 construct facilitates optimal graft integration and minimizes tunnel widening. Also, this technique preserves the gracilis tendon, potentially reducing knee flexion strength deficits. The DSTG technique may result in a greater internal rotation deficit compared to the ST4 technique, with the latter resulting in less active flexion range and strength loss [46, 47]. The good clinical results of both techniques concerning knee laxity and high knee function at PROMs are in concert with other studies comparing clinical outcomes between all-inside and traditional techniques [48].

A key clinical outcome after ACLR is return to sport (RTS). How demonstrated by a recent systematic review by D’Ambrosi et al., however, conventional MRI-derived markers (graft bending angle, SNQ/SNR, qualitative signal grades/Howell score) show no reliable correlation with RTS, indicating that MRI should presently be considered adjunctive rather than determinative for RTS decisions. [49]

This study has several limitations. First, allocation was sequential and non-randomized, guided by surgeon scheduling and implant availability. Although baseline characteristics were comparable, residual selection bias and unmeasured confounding cannot be excluded. Second, the single-center, single-surgeon design may limit generalizability and introduces the possibility of a technique-specific learning curve. Third, MRI was obtained only at 12 months, so temporal trajectories of graft signal and tunnel remodeling beyond this time point are unknown. Fourth, SNQ is a sequence-dependent surrogate biomarker; despite using a uniform PD-FS protocol and standardized measurements with good reliability, the biological significance of small SNQ differences, and their relationship to graft biomechanics, remains uncertain. Fifth, while tibial tunnel widening differed between techniques, this radiologic finding did not translate into superior clinical outcomes at 24 months, and the study was not powered to detect small between-group differences in PROMs. Sixth, adherence to rehabilitation was not objectively monitored and could have influenced imaging or clinical endpoints. Finally, an a priori sample size for non-inferiority indicated an impractically large sample (1,356 participants) would be required to ensure a non-inferiority statement; accordingly, a non-inferiority analysis was not conducted.

## Conclusions

AI ST4 and DSTG ACL reconstruction demonstrated similar graft maturation at 12 months, with less tibial tunnel widening after AI–ST4. At 24 months, clinical outcomes were equivalent between groups; although SNQ and tibial tunnel CSA demonstrated good reproducibility, these imaging differences were not demonstrably associated with functional superiority.

## Data Availability

De-identified individual participant data (IPD), imaging measurement worksheets, and analysis scripts are available from the corresponding author upon reasonable request and subject to institutional data-sharing policies and GDPR limitations.
